# More comprehensive forensic genetic marker analyses for accurate human remains identification using massively parallel DNA sequencing

**DOI:** 10.1186/s12864-016-3087-2

**Published:** 2016-10-17

**Authors:** Angie D. Ambers, Jennifer D. Churchill, Jonathan L. King, Monika Stoljarova, Harrell Gill-King, Mourad Assidi, Muhammad Abu-Elmagd, Abdelbaset Buhmeida, Bruce Budowle

**Affiliations:** 1 Institute of Applied Genetics, Department of Molecular and Medical Genetics, University of North, Texas Health Science Center, 3500 Camp Bowie Boulevard, Fort Worth, TX USA; 2 Institute of Gene Technology, Department of Molecular Diagnostics, Tallinn University of Technology, Akadeemia tee 15A-604, Tallinn, 12618 Estonia; 3 Department of Biological Sciences, Laboratory of Forensic Anthropology, Center for Human Identification, University of North Texas, 1511 W. Sycamore, Denton, TX USA; 4 Center of Excellence in Genomic Medicine Research (CEGMR), King Abdulaziz University, Jeddah, Saudi Arabia

**Keywords:** Human skeletal remains, Massively parallel sequencing (MPS), Phenotype-informative SNPs, Y-STRs, Mitochondrial DNA, Ancestry-informative markers (AIMS)

## Abstract

**Background:**

Although the primary objective of forensic DNA analyses of unidentified human remains is positive identification, cases involving historical or archaeological skeletal remains often lack reference samples for comparison. Massively parallel sequencing (MPS) offers an opportunity to provide biometric data in such cases, and these cases provide valuable data on the feasibility of applying MPS for characterization of modern forensic casework samples. In this study, MPS was used to characterize 140-year-old human skeletal remains discovered at a historical site in Deadwood, South Dakota, United States. The remains were in an unmarked grave and there were no records or other metadata available regarding the identity of the individual. Due to the high throughput of MPS, a variety of biometric markers could be typed using a single sample.

**Results:**

Using MPS and suitable forensic genetic markers, more relevant information could be obtained from a limited quantity and quality sample. Results were obtained for 25/26 Y-STRs, 34/34 Y SNPs, 166/166 ancestry-informative SNPs, 24/24 phenotype-informative SNPs, 102/102 human identity SNPs, 27/29 autosomal STRs (plus amelogenin), and 4/8 X-STRs (as well as ten regions of mtDNA). The Y-chromosome (Y-STR, Y-SNP) and mtDNA profiles of the unidentified skeletal remains are consistent with the R1b and H1 haplogroups, respectively. Both of these haplogroups are the most common haplogroups in Western Europe. Ancestry-informative SNP analysis also supported European ancestry. The genetic results are consistent with anthropological findings that the remains belong to a male of European ancestry (Caucasian). Phenotype-informative SNP data provided strong support that the individual had light red hair and brown eyes.

**Conclusions:**

This study is among the first to genetically characterize historical human remains with forensic genetic marker kits specifically designed for MPS. The outcome demonstrates that substantially more genetic information can be obtained from the same initial quantities of DNA as that of current CE-based analyses.

**Electronic supplementary material:**

The online version of this article (doi:10.1186/s12864-016-3087-2) contains supplementary material, which is available to authorized users.

## Background

The paramount goal of forensic DNA testing of human skeletal remains is identification of the unknown individual. A variety of genetic markers can be used to achieve identification, including highly polymorphic autosomal short tandem repeat (STR) loci and lineage markers [Y-STRs, Y chromosome single nucleotide polymorphisms (Y-SNPs), mitochondrial DNA (mtDNA)]. However, reference samples must be available for comparison for these markers to be informative. In mass disasters, missing persons cases, or cases involving historical/archaeological remains, sometimes there are no clues as to the person’s potential identity and/or there are no associations made with a reference sample or reference pedigree via a database search [1]. In such scenarios, identification can be difficult or impossible using solely autosomal STRs and lineage markers. However, there are other genetic markers that can extend human identification capabilities, such as analysis of ancestry-informative markers [2–7] and phenotype-informative SNPs [7–11].

Massively parallel sequencing (MPS) of ancestry- and phenotype-informative SNPs, with its expanded capacity for marker typing, offers the ability to develop investigative leads in such cases [12–16]. Thus, more genetic information can be gleaned from a sample without further consumption of often very limited quantity and quality samples. In this study, MPS was used in an effort to help characterize 140-year-old human skeletal remains that were buried in an unmarked grave in Deadwood, South Dakota USA, a famous town of the American Old West.

In 1874, the discovery of gold in the Black Hills of South Dakota set off one of the last great gold rushes in America. In 1876, miners moved to the area and formally established the city of Deadwood, now a U.S. historical landmark. Deadwood’s original cemetery, Ingleside, was located near the town’s core business district and contained approximately 100 burials (although cemetery records are incomplete and some were buried in unmarked graves). In 1878, the individuals interred there were relocated to the hills above Deadwood, and Mount Moriah Cemetery was established.

In 2012, a set of unidentified human skeletal remains were unearthed by a construction crew in Deadwood’s Presidential District, the original site of Ingleside Cemetery [17–19]. South Dakota State archaeologists and historic preservation officials for the City of Deadwood recovered the skeleton from the site (with the exception of one tooth and a few bones from the hands and feet). Anthropological analyses indicated that the remains are consistent with a male of European ancestry (Caucasian) who was 18–24 years of age at the time of death and 65.7 − 70.7 inches tall. No indications of the cause of death were evident in the skeletal samples [19–21]. Forensic odontological analyses determined that this unknown individual was a habitual tobacco user and had nine dental fillings (3 gold, 6 tin/amalgam). The latter observation is indicative of some level of affluence/wealth, as most individuals in the late 19^th^ century would simply have had unhealthy teeth extracted [20, 21].

In June 2014, the City of Deadwood and the Deadwood Historic Preservation Commission requested that the Institute of Applied Genetics (IAG) conduct DNA testing on the remains to provide some level of identification [18–21]. Given that the remains were in an unmarked grave and no investigative leads existed regarding his identity, Deadwood city officials were interested in the analysis of DNA markers that could help predict the individual’s ancestry and external physical traits. Markers chosen for analysis included Y-STRs, Y-SNPs, ancestry-informative SNPs, phenotype-informative SNPs, and mitochondrial DNA (mtDNA). To the best of our knowledge, this study is among the first to genetically characterize historical human remains with forensic genetic marker kits specifically designed for MPS.

## Methods

The practices for minimizing contamination during the analysis of the Deadwood remains were the same contamination controls recommended for archaeological and ancient DNA specimens, including: (a) use of protective suits, gloves, and masks; (b) bleach de-contamination and UV-irradiation of work benches and associated equipment; (c) physical removal and/or chemical destruction of contaminant/exogenous DNA on external bone surfaces; (d) extraction of bone samples in a designated low-copy area; (e) PCR amplification in a location that is physically separated from the extraction area; (f) use of appropriate negative controls, reagent blanks, and positive controls; and (g) replicate testing [22–28].

### Bone processing and DNA extraction

**Fig. 1:**
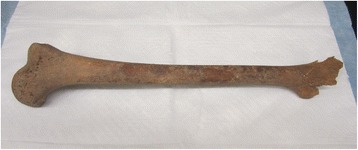
Right femur of unidentified human skeletal remains discovered in 2012 in Deadwood’s Presidential District

The right femur was provided to the IAG for DNA testing (Loan Accession No. 12–0051, South Dakota Archaeological Research Center) (Fig. 1). A portion of the femur diaphysis was surface-sanded with a Dremel® 4000 Rotary Tool and sterile grinding stone (Robert Bosch Tool Corporation, Mount Prospect, Illinois USA) followed by sectioning of eight adjacent regions with a Stryker® autopsy saw (Mopec, Oak Park, Michigan USA). DNA extractions were performed on six of the eight bone sections in a designated low-copy number (LCN) area of the laboratory, as described in Ambers et al. [29].

### DNA quantification

The quantity of DNA from seven bone powder fractions was determined using the Quantifiler® Human DNA Quantification Kit and an ABI 7500 Real-Time PCR System (Thermo Fisher Scientific, Waltham, Massachusetts USA), according to manufacturers’ recommendations [30].

### Traditional Y-STR typing via capillary electrophoresis

Human genomic DNA was amplified with reagents from the AmpFlSTR® Yfiler™ PCR Amplification Kit and a GeneAmp® PCR System 9700 (Thermo Fisher Scientific), according to manufacturer’s recommendations [31]. Negative controls consisted of 10 μl low-TE buffer and 10 μl 9947A female DNA (0.1 ng/μl); 10 μl 007 Male Control DNA (0.1 ng/μl) served as the positive control. PCR products were separated via capillary electrophoresis (CE) on a 3500*xl* Genetic Analyzer, and analyzed using GeneMapper® ID-X software (Thermo Fisher Scientific). DNA (elution #1 and elution #2) from seven bone powder fractions was typed.

### Massively Parallel Sequencing (MPS) with the Illumina MiSeq®

DNA from four of the bone powder extracts (007.001 E1, 008.001 E1, 008.002 E1, 008.002 E2) that yielded partial to complete Yfiler™ Y-STR profiles was analyzed via MPS. The beta version of the ForenSeq™ DNA Signature Prep Kit (Illumina, San Diego, California USA) was used to prepare libraries as described in [32]. For the Illumina® ForenSeq DNA Signature Prep Kit, the Y-STR markers analyzed were: DYF387S1, DYS19, DYS385a/b, DYS389I, DYS389II, DYS390, DYS391, DYS392, DYS437, DYS438, DYS439, DYS448, DYS456, DYS460, DYS481, DYS505, DYS522, DYS533, DYS549, DYS570, DYS576, DYS612, DYS635, DYS643, and Y-GATA H4. Input DNA was 0.20 ng, 1 ng, 1 ng, and 0.58 ng, respectively, for the first PCR. Ten microliters of pooled libraries were used for the proceeding “Denature and Dilute Libraries” step. Subsequent sequencing on the MiSeq® Desktop Sequencer (Illumina) and data analysis were completed as detailed in [32].

### Massively Parallel Sequencing (MPS) with the Ion Torrent PGM®

DNA from three of the same four bone extracts (008.001 E1, 008.002 E1, 008.002 E2) was analyzed on the Ion Torrent Personal Genome Machine® (PGM) platform (Thermo Fisher Scientific). Library preparation, sequencing, and data analysis for three SNP panels [HID-Ion AmpliSeq™ Identity Panel, HID-Ion AmpliSeq™ Ancestry Panel, and an Externally Visible Characteristics (EVC) prototype panel (Thermo Fisher Scientific)] were completed as described in [33–36]. Input DNA was 1 ng, 1 ng, and 0.58 ng, respectively, 22 cycles were used in the initial PCR, and 25 μl of pooled libraries were used for preparation of the Ion OneTouch™ 2 (OT2) amplification solution. Mitochondrial DNA was amplified using an in-house PCR multiplex assay [unpublished]. Eight positions of the mtDNA coding region were sequenced: 4488–4656, 4727–4862, 8542–8707, 10674–10830, 13588–13745, 13809–14098, 14133–14301, and 14766–14923. The noncoding hypervariable regions (HVI, HVII) also were sequenced, as described in [37]. Library preparation, sequencing, and data analysis were completed as outlined in [36] with one exception: 25 μl of pooled libraries were used for preparation of the OT2 amplification solution.

### Final data analysis

30X and 10X coverage were set as minimum detection thresholds for the autosomal markers and mtDNA typed by MPS in this study, respectively. The Y haplogroup was determined using the ancestry feature and metapopulation tool of the Y-STR haplotype reference database YHRD (www.yhrd.org). A PCA plot of ancestry-informative SNP data was generated with the Illumina® ForenSeq™ Universal Analysis Software. Mitochondrial DNA sequence alignment was performed with the mitoSAVE workbook [38], and haplogroup determination was made using HaploGrep software (http://haplogrep.uibk.ac.at/) [39]. Phenotypic SNP data were analyzed with the Illumina® ForenSeq™ Universal Analysis Software as well as with the HIrisplex hair/eye color prediction tool (http://hirisplex.erasmusmc.nl) [9, 10].

## Results and discussion

DNA concentrations recovered from the right femur powder fractions ranged from 0.0147–0.3350 ng/μl for elution #1 and 0–0.0579 ng/μl for elution #2, respectively. The elution volume for each DNA extract was 30 μl, and the total DNA recovered per elution is reported in Table 1.

**Table 1:** DNA concentrations (ng/μl) obtained from the right femur of Deadwood’s unidentified human skeletal remains (E1 = elution #1; E2 = elution #2; total elution volume = 30 μl)

Sample ID	ng/μl	Total DNA (ng)
Femur 001.001 E1	0.0327	0.98
Femur 001.001 E2	0.0082	0.24
Femur 002.002 E1	0.0232	0.70
Femur 002.002 E2	0.0029	0.09
Femur 003.001 E1	0.0147	0.44
Femur 003.001 E2	undetermined	undetermined
Femur 006.002 E1	0.1730	5.19
Femur 006.002 E2	0.0147	0.44
Femur 007.001 E1	0.0383	1.15
Femur 007.001 E2	0.0014	0.04
Femur 008.001 E1	0.3080	9.24
Femur 008.001 E2	0.0347	1.04
Femur 008.002 E1	0.3350	10.05
Femur 008.002 E2	0.0579	1.74

A variety of STR and SNP markers were analyzed via CE and MPS. No DNA was detected in any of the negative controls and reagent blanks, and positive controls yielded the correct type for all analyses.

### Y-chromosome (Y-STR and Y-SNP) DNA Analysis: CE and MPS

Y-STR typing results varied among the samples (Tables 2 and 3). Allele calls among all extracts were concordant. Individual sample results and the complete 17-locus Yfiler™ consensus profile are shown in Table 2. Fifteen of the twenty-six Y-STR markers analyzed with the Illumina® ForenSeq™ DNA Signature Prep Kit overlap with the AmpFlSTR® Yfiler™ PCR Amplification Kit. The Y-STR alleles recovered from all bone samples among the common markers between MPS and CE were concordant. Y-STR typing results were obtained for 17 of the 26 markers assayed with MPS (Table 3); coverage ranged from 31x to 620x [148 ± 137 (Avg ± SD)]. The total number of Y-STR loci that yielded results for both methods was 25.

**Table 2:** Y-STR typing allele results for 17 loci in seven different bone powder fractions using the AmpFlSTR® Yfiler™ PCR Amplification Kit and CE (E1 = elution #1; E2 = elution #2)

Sample Name	DYS456	DYS389 I	DYS390	DYS389 II	DYS458	DYS19	DYS385 a/b	DYS393	DYS391	DYS439	DYS635	DYS392	Y GATA H4	DYS437	DYS438	DYS448
FEMUR 001.001 E1																
FEMUR 001.001 E2																
FEMUR 002.002 E1														15		
FEMUR 002.002 E2								13						15		
FEMUR 003.001 E1																
FEMUR 003.001 E2																
FEMUR 006.002 E1								13	11					15		
FEMUR 006.002 E2	16	14			19			13	11					15		
FEMUR 007.001 E1	16	14			19			13	11					15		
FEMUR 007.001 E2									11							
FEMUR 008.001 E1	16	14	24	30	19	14	11,14	13	11	11	23	13	12	15	12	19
FEMUR 008.001 E2	16	14			19		11		11	11	23		12	15	12	19
FEMUR 008.002 E1	16	14	24	30	19	14	14	13	11	11	23		12	15	12	19
FEMUR 008.002 E2	16	14	24		19	14		13	11	11	23		12	15	12	19
Consensus Y-STR Haplotype:	16	14	24	30	19	14	11,14	13	11	11	23	13	12	15	12	19

**Table 3:** Y-STR typing allele results for four different bone powder fractions using the Illumina® ForenSeq™ DNA Signature Prep Kit and MPS (E1 = elution #1; E2 = elution #2; markers in common with AmpFlSTR® Yfiler™ are shown in **bold**)

Sample Name	DYF387S1	DYS19	DYS385a-b	DYS389I	DYS389II	DYS390	DYS391	DYS392	DYS437	DYS438	DYS439	DYS448	DYS456	DYS460	DYS481	DYS505	DYS522	DYS533	DYS549	DYS570	DYS576	DYS612	DYS635	DYS643	Y-GATA H4
FEMUR 007.001 E1										**12**					22										
FEMUR 008.001 E1				**14**			**11**		**15**	**12**	**11**				22	12				17	17			10	
FEMUR 008.002 E1	36			**14**			**11**		**15**	**12**	**11**				22	12				17	17	39		10	
FEMUR 008.002 E2	36	**14**		**14**			**11**			**12**	**11**	**19**			22		10	12					**23**	10	
Consensus Y-STR Haplotype	36	**14**		**14**			**11**		**15**	**12**	**11**	**19**			22	12	10	12		17	17	39	**23**	10	

The composite 17-locus Y-STR profile generated with AmpFlSTR® Yfiler™ and the additional Y STR loci from the Illumina® ForenSeq™ DNA Signature Prep Kit is consistent with the R1b haplogroup. R1b is the most common Y haplogroup in Western Europe, spanning 80 % of the population in Ireland, western Wales, the Scottish Highlands, the Atlantic fringe of France, Catalonia, and the Basque country. It also is common around the Caucasus and in Anatolia, in parts of Russia, and in Central and South Asia [40–45].

In addition to Y-STR data, a consensus Y-SNP profile was compiled using data from three different bone powder fractions from the Deadwood unidentified skeletal remains. All 34 upper clade Y-SNPs in the HID-Ion AmpliSeq™ Identity Panel provided typing results [Additional file 1], and these haplogroup-informative Y-SNP results also supported an R1b haplogroup assignment.

### Ancestry informative SNPs

**Fig. 2:**
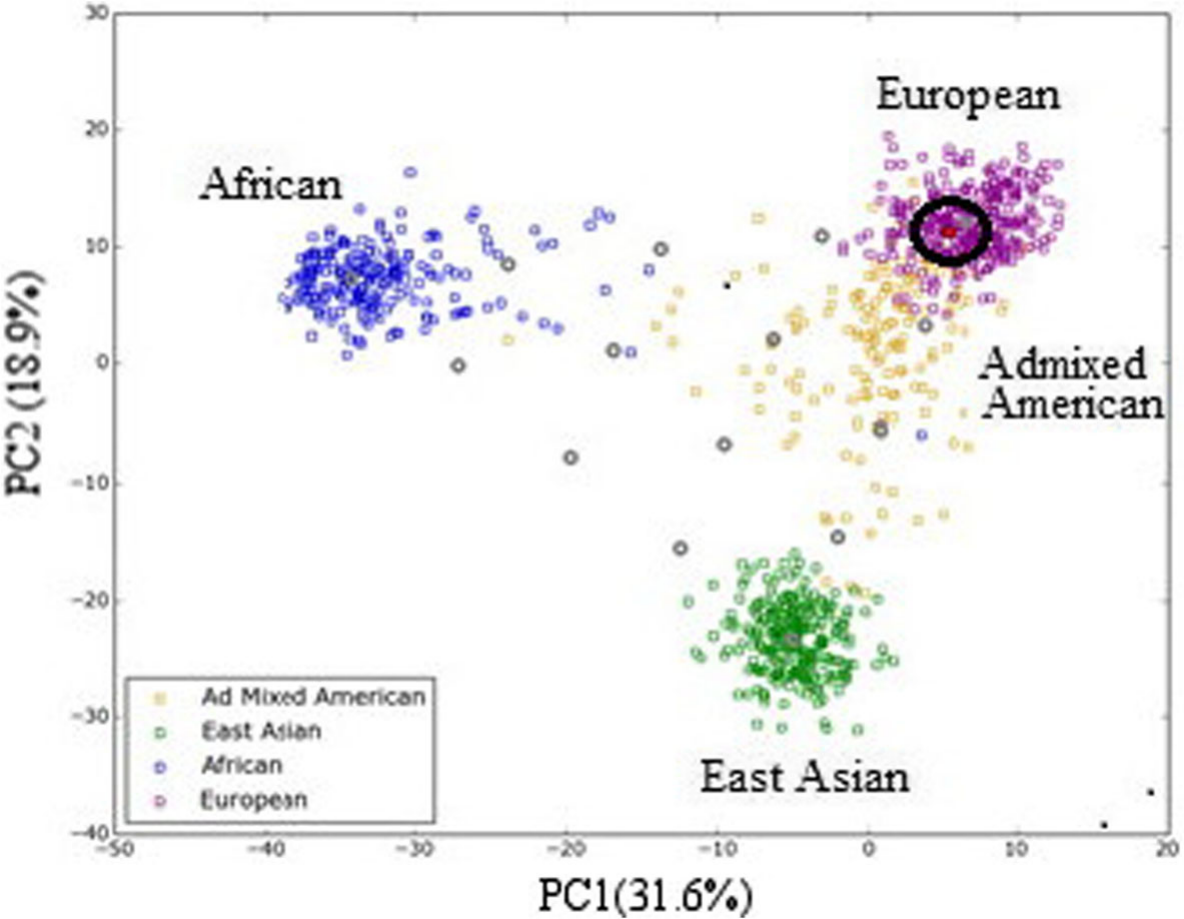
PCA plot of ancestry-informative SNPs, displaying the best fit population assignment. The population assignment for the 140-year-old unidentified Deadwood skeletal remains is circled in the scatter plot

Ancestry-informative SNP results were obtained for 51 of the 54 SNP markers amplified via the Illumina® ForenSeq™ DNA Signature Prep Kit, and for all 165 markers tested using the HID-Ion AmpliSeq™ Ancestry Panel. Depth of coverage ranged from 31x to 2240x (170 ± 107) and 53x to 1190x (379 ± 243), respectively. Fifty-three of the ancestry-informative SNPs in the Illumina® ForenSeq™ kit are included in the HID-Ion AmpliSeq™ Ancestry Panel, and 51 of these SNPs yielded results with both panels. The results were concordant, and a composite profile was generated [Additional file 2]. Using the ancestry-informative SNP data, the major population bio-ancestry was determined to be European (Fig. 2).

### Mitochondrial DNA (mtDNA) Analysis

An in-house PCR multiplex assay comprised of short amplicons (~200 bp in length) at targeted sites on the coding and non-coding regions (HVI and HVII) of the mitochondrial DNA (mtDNA) genome was used to characterize the maternal lineage of the Deadwood skeletal remains. A total of 10 regions of the mtDNA genome were assayed (HVI, HVII, and eight coding regions covering 2120 bases). The mtDNA regions targeted by the in-house multiplex assay increase discrimination power by 17.4 % beyond sequencing of HV1 and HVII alone (unpublished data). Quality sequencing results were obtained for all 10 regions, with a range in coverage of 19x to 23,987x for 95.3 % of the targeted regions. Nine of the 10 regions sequenced had greater than 1000x coverage (14133–14301 had an average coverage of ~100x) (Additional file 3). No inference of heteroplasmy was observed.

The reportable mtDNA data from these ten regions (2020 bp; 30–305, 4488–4656, 4707–4880, 8520–8726, 10657–10847, 13570–13760, 13791–13990, 14133–14301, 14749–14941, 15980–16407) allowed for the following haplotype to be constructed for the reported region (146C, 263G, 4769G, 16181G, 16183C, 16189C). These data provided sufficient genetic information to determine that the most likely mtDNA haplogroup is H1 with numerous subgroups of H1 (e.g., H1 + 16189, H1f, H1y, etc.) giving 83.2 % quality score via HaploGrep [39]. Mitochondrial haplogroup H1 is the most common in Western Europe and is found throughout Europe, northern Africa, the Levant, the Caucasus, Anatolia, and as far as Central Asia and Siberia [46–52]. Hence, the biogeographic ancestry determined by the Y-STR, Y-SNP, ancestry-informative SNP, and mtDNA data are all consistent with that obtained by anthropological analyses of a European ancestry.

### Forensic DNA phenotyping

Twenty-four phenotype-informative SNPs were assayed using the Ion Torrent PGM® and HID-Ion AmpliSeq™ Externally Visible Characteristics (EVC) Prototype Panel. Results were obtained for 23 of the 24 phenotype-informative SNPs assayed, with a depth of coverage of 33x to 1419x (282 ± 205) (Table 4).

**Table 4:** Phenotype-informative SNP analysis results for three Deadwood skeletal samples using the Ion Torrent PGM® and HID-Ion AmpliSeq™ Externally Visible Characteristics (EVC) Prototype Panel

Sample Name	rs12896399	rs28777	rs1393350	rs16891982	rs12203592	N29insA	rs1042602	rs2378249	rs12821256	rs1800407	rs12913832	rs683	rs2402130	rs4959270	rs1805005	rs1805006	rs2228479	rs1110400	rs11547464	rs1805007	rs1805008	rs1805009	Y152OCH	rs2228479
Femur 008.001 E1	G/G	A/A	G/G	G/G		C/C	C/C	G/A	T/T	G/G	A/G	C/A	A/A	C/A	G/G	C/C	G/G	T/T	G/G	C/C	T/T	G/G	C/C	G/G
Femur 008.002 E1	G/G	A/A	G/G	G/G		C/C	C/C	G/A	T/T	G/G	A/G	C/A	A/A	C/A	G/G	C/C	G/G	T/T	G/G	C/C	T/T	G/G	C/C	G/G
Femur 008.002 E2		A/A	G/G	G/G		C/C		G/A	T/T	G/G	A/G	C/A	A/A	C/A	G/G	C/C	G/G	T/T	G/G	C/C	T/T		C/C	G/G

Additional testing was performed on the skeletal samples using the Illumina® ForenSeq™ DNA Signature Prep Kit and MiSeq® platform. Results were obtained for all 24 phenotype-informative SNP markers assayed, with a depth of coverage of 32x to 1187x (288 ± 407). Typing results were concordant between assays and between the two MPS platforms. A composite phenotype-informative SNP profile was generated and is shown in Additional file 4. Phenotypic SNP analysis was performed using the HIrisPlex hair/eye color prediction tool (http://hirisplex.erasmusmc.nl), which generates individual probabilities for four hair color categories (red, blonde, brown, black), two hair color shades (light, dark), and three eye color categories (blue, intermediate, brown) [9, 10]. The 24 predictive DNA variants (23 SNPs and 1 INDEL) of the HIrisPlex assay are included in the Illumina® ForenSeq™and HID-Ion AmpliSeq™ Externally Visible Characteristics (EVC) Prototype Panel, and the system was designed to cope with low template and degraded DNA. All 24 DNA variants have small amplicon sizes (< 160 bp). In terms of specificity, HIrisPlex variants provide blue and brown human eye color predictions with over 90 % precision [9] and average hair color prediction accuracies of 0.70, 0.79, 0.80, and 0.88 for red, blonde, brown, and black hair, respectively [10]. Analysis of the Deadwood skeletal remains indicated that this individual likely had light red hair and light brown eyes. Probabilities for hair color, hair color shade, and eye color were 0.69, 0.71, and 0.72, respectively (Table 5).

**Table 5:** Prediction of Deadwood undentified skeletal remains’ potential hair and eye color using phenotype-informative SNP data and the HIrisplex hair/eye color prediction tool (http://hirisplex.erasmusmc.nl/) [10]

HAIR COLOR		HAIR SHADE		EYE COLOR	
Brown	0.19	Light	0.71	Brown	0.72
Red	0.69	Dark	0.29	Intermediate	0.19
Black	0.04			Blue	0.09
Blonde	0.09				

### Other markers assayed with MPS panels

The Illumina® ForenSeq™ DNA Signature Prep Kit and the HID-Ion AmpliSeq™ Identity Panel also contain markers that do not contribute to the characterization of bioancestry or phenotype, but nonetheless were able to be typed. With the Illumina® ForenSeq™ DNA Signature Prep Kit, results were obtained for 88/95 human identity SNPs, 27/29 autosomal STRs (plus amelogenin), and 4/8 X-STRs. Range in coverage for the human identity SNPs, autosomal STRs, and X-STRs were 32x-1085x (217 ± 213), 31x-2838x (297 ± 485), and 31x-361x (170 ± 107), respectively. With the HID-Ion AmpliSeq™ Identity Panel, results were obtained for 90/90 human identity SNPs [Additional files 5, 6 and 7], with a depth of coverage of 33x-1419x (282 ± 205). There are 80 human identity SNPs in common between the two kits, and 75 of these common markers yielded results with both panels. Results were concordant between the two identity SNP panels. These results further support the potential of MPS to enable typing of a much larger number of genetic markers from the same amount of DNA than would have been possible with current CE-based systems.

## Conclusion

In an effort to learn more about the late-19^th^-century human skeletal remains discovered at the site of Deadwood’s first cemetery, historic preservation officials enlisted a number of forensic specialists to conduct analyses on the remains that could assist in his identification [17–21, 53]. Since the individual was buried in an unmarked grave and no investigative leads existed regarding his identity, lineage testing and forensic DNA phenotyping were performed to predict ancestry and external physical traits.

The Y-chromosome (Y-STR, Y-SNP) and mitochondrial DNA (mtDNA) profiles of the unidentified skeletal remains are consistent with the R1b and H1 haplogroups, respectively. Both of these haplogroups are the most common ones in Western Europe. The ancestry-informative SNPs also indicated a European background. These genetic results are consistent with the findings of a previous anthropological report which determined that the Deadwood unidentified skeletal remains belong to a male of European ancestry (Caucasian). The phenotype-informative SNPs provided strong support that the individual had light red hair and brown eyes. This study is among the first known historical remains case that has been characterized with genetic panels designed specifically for forensic human identification purposes. The results were highly informative. The study demonstrates the potential of MPS to analyze unidentified human skeletal remains and to provide substantially more genetic information from the same initial quantities of DNA sample as that of CE-based analyses. Using the Illumina® ForenSeq™ DNA Signature Prep Kit, results were obtained for 25/26 Y-STRs, 88/95 human identity SNPs, 51/54 ancestry-informative SNPs, 24/24 phenotype-informative SNPs, 27/29 autosomal STRs (plus amelogenin), and 4/8 X-STRs. With the HID-Ion AmpliSeq™ Identity Panel, results were obtained for 34/34 Y-SNPs and 90/90 human identity SNPs. The HID-Ion AmpliSeq™ Ancestry Panel yielded data for 165/165 ancestry-informative SNP markers assayed. Combined results for all MPS panels included genetic data for 25/26 Y-STRs, 34/34 Y SNPs, 166/166 ancestry-informative SNPs, 24/24 phenotype-informative SNPs, 102/102 human identity SNPs, 27/29 autosomal STRs (plus amelogenin), and 4/8 X-STRs (as well as ten regions of mtDNA).

An important point about DNA testing of historical or archaeological skeletal remains should be emphasized. Six bone sections/cuttings were taken, and bone powder fractions from each were analyzed. Adjacent bone sections yielded vastly different results in terms of DNA quantity and number of allele calls; some regions of bone did not yield any DNA, while other areas yielded complete profiles. These findings are consistent with a previous study performed on the 120-year-old skeletal remains of an American Civil War soldier [29], which required testing of multiple bone sections and a consensus testing approach to obtain a complete Y-STR haplotype.

With its capacity for simultaneous analysis of a multitude of different types of DNA markers, MPS technology holds promise for use in the characterization of historical and archaeological remains, and in missing persons cases. In addition, in mass disasters or other types of cases where reference samples are not available/known, genetic markers such as ancestry-informative and phenotype-informative SNPs can provide data for craniofacial reconstructions that could be useful for positive identification.

## Additional files

Additional file 1: Y-SNP data for three different bone powder fractions using the HID-Ion AmpliSeq™ Identity Panel and the Ion Torrent PGM® MPS platform (E1 = elution #1; E2 = elution #2). (XLSX 12 kb)
Additional file 2: Ancestry-informative SNP results for 140-year-old skeletal remains from Deadwood, Dakota using the A) Illumina® ForenSeq™ panel and B) HID-Ion AmpliSeq™ Ancestry Panel. Concordant results for the 53 ancestry-informative SNPs that were common between the two assays were used to generate a composite AIMS profile (C). (XLSX 20 kb)
Additional file 3: Sequence results for ten regions of the mtDNA genome using an in-house mtDNA panel (unpublished). The overall coverage ranged from 0-23987x. The lowest coverage (~100x) region (14133–14301) is indicated with black box and arrow. A) 140-year-old skeletal remains, B) reagent blank, and C) positive control. (JPG 136 kb)
Additional file 4: Phenotype-informative SNP analysis results for 140-year-old skeletal remains from Deadwood, Dakota using the: A) Illumina® ForenSeq™ DNA Signature Prep Kit and B) HID-Ion AmpliSeq™ Externally Visible Characteristics (EVC) Prototype Panel (E1 = elution #1; E2 = elution #2). Concordant results were used to generate a composite profile (C). (XLSX 12 kb)
Additional file 5: Human identity SNP results for 140-year-old skeletal remains from Deadwood, Dakota using the: A) Illumina® ForenSeq™ DNA Signature Prep Kit and B) HID-Ion AmpliSeq™ Identity Panel (E1 = elution #1; E2 = elution #2). Eighty of the human identity SNPs were common between the two panels (shown in bold). Concordant results were used to generate a composite profile (C). (XLSX 17 kb)
Additional file 6: Autosomal STR typing results for 140-year-old skeletal remains from Deadwood, Dakota using the Illumina® ForenSeq™ DNA Signature Prep Kit and MiSeq® MPS platform (E1 = elution #1; E2 = elution #2). (XLSX 12 kb)
Additional file 7: X-STR typing results for 140-year-old skeletal remains from Deadwood, South Dakota using the Illumina® ForenSeq™ DNA Signature Prep Kit and MiSeq® MPS platform (E1 = elution #1; E2 = elution #2). (XLSX 11 kb)
