# Erratum to: More Comprehensive Forensic Genetic Marker Analyses for Accurate Human Remains Identification Using Massively Parallel DNA Sequencing

**DOI:** 10.1186/s12864-017-3648-z

**Published:** 2017-04-20

**Authors:** Angie D. Ambers, Jennifer D. Churchill, Jonathan L. King, Monika Stoljarova, Harrell Gill-King, Mourad Assidi, Muhammad Abu-Elmagd, Abdelbaset Buhmeida, Mohammed Al-Qahtani, Bruce Budowle

**Affiliations:** 1 Institute of Applied Genetics, Department of Molecular and Medical Genetics, University of North Texas Health Science Center, 3500 Camp Bowie Boulevard, Fort Worth, Texas USA; 2 Institute of Gene Technology, Department of Molecular Diagnostics, Tallinn University of Technology, Akadeemia tee 15A-604, Tallinn, 12618 Estonia; 3 Laboratory of Forensic Anthropology, Center for Human Identification, University of North Texas, Department of Biological Sciences, 1511 W. Sycamore, Denton, Texas USA; 4 Center of Excellence in Genomic Medicine Research (CEGMR), King Abdulaziz University, Jeddah, Saudi Arabia

## Erratum

This article [1] unfortunately published with an author deleted in the author list. The correct author list is presented above.

The author list was previously incorrectly presented as:

Angie D. Ambers^1*^, Jennifer D. Churchill^1^, Jonathan L. King^1^, Monika Stoljarova^1,2^, Harrell Gill-King^3^, Mourad Assidi^4^, Muhammad Abu-Elmagd^4^, Abdelbaset Buhmeida^4^ and Bruce Budowle^1,4*^

^1^Institute of Applied Genetics, Department of Molecular and Medical Genetics, University of North, Texas Health Science Center, 3500 Camp Bowie Boulevard, Fort Worth, TX, USA. ^2^Institute of Gene Technology, Department of Molecular Diagnostics, Tallinn University of Technology, Akadeemia tee 15A-604, Tallinn 12618, Estonia. ^3^Department of Biological Sciences, Laboratory of Forensic Anthropology, Center for Human Identification, University of North Texas, 1511 W. Sycamore, Denton, TX, USA. ^4^Center of Excellence in Genomic Medicine Research (CEGMR), King Abdulaziz University, Jeddah, Saudi Arabia.
