# Supplementary figures and images for: More comprehensive forensic genetic marker analyses for accurate human remains identification using massively parallel DNA sequencing
Source: BMC Genomics. 2016 Oct 17;17(Suppl 9):750. doi: 10.1186/s12864-016-3087-2 (PMC5073988; doi:10.1186/s12864-016-3087-2)

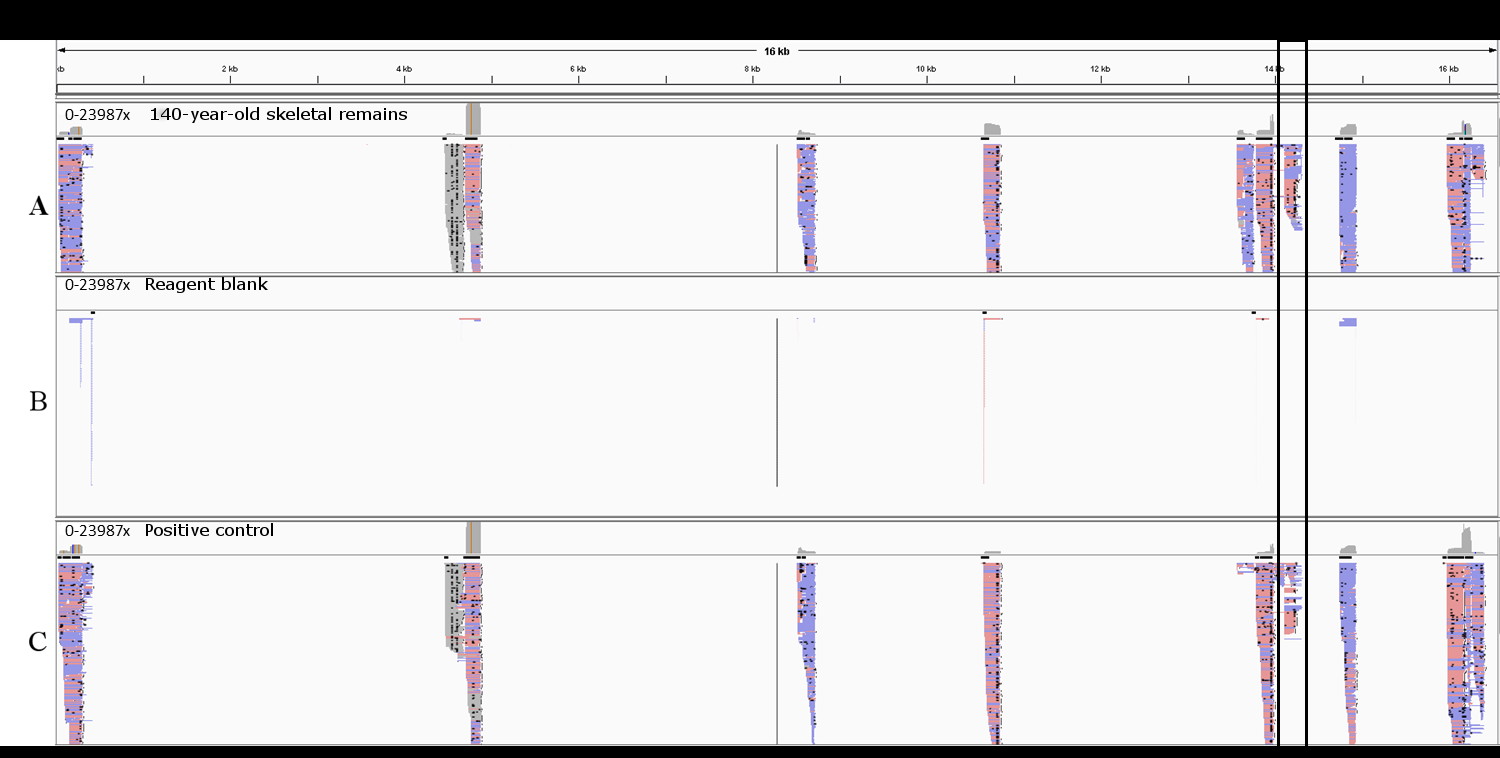

Supplement: Additional file 3: — Sequence results for ten regions of the mtDNA genome using an in-house mtDNA panel (unpublished). The overall coverage ranged from 0-23987x. The lowest coverage (~100x) region (14133–14301) is indicated with black box and arrow. A) 140-year-old skeletal remains, B) reagent blank, and C) positive control. (JPG 136 kb) [file 12864_2016_3087_MOESM3_ESM.jpg]
